# Is Sustained Virological Response a Marker of Treatment Efficacy in Patients with Chronic Hepatitis C Viral Infection with No Response or Relapse to Previous Antiviral Intervention?

**DOI:** 10.1371/journal.pone.0083313

**Published:** 2013-12-12

**Authors:** Kurinchi S. Gurusamy, Edward Wilson, Ronald L. Koretz, Victoria B. Allen, Brian R. Davidson, Andrew K. Burroughs, Christian Gluud

**Affiliations:** 1 Department of Surgery, University College London, London, United Kingdom; 2 Health Economics Group, University of East Anglia, Norwich, United Kingdom; 3 Cambridge Centre for Health Services Research, University of Cambridge, Cambridge, United Kingdom; 4 Cochrane Hepato Biliary Group, Granada Hills, California, United States of America; 5 Sheila Sherlock Liver Centre and Institute of Liver and Digestive Health, Royal Free Hospital, and UCL, London, United Kingdom; 6 Cochrane Hepato-Biliary Group, The Copenhagen Trial Unit, Centre for Clinical Intervention Research, Rigshospitalet,Copenhagen University Hospital, Copenhagen, Denmark; Saint Louis University, United States of America

## Abstract

**Background:**

Randomised clinical trials (RCTs) of antiviral interventions in patients with chronic hepatitis C virus (HCV) infection use sustained virological response (SVR) as the main outcome. There is sparse information on long-term mortality from RCTs.

**Methods:**

We created a decision tree model based on a Cochrane systematic review on interferon retreatment for patients who did not respond to initial therapy or who relapsed following SVR. Extrapolating data to 20 years, we modelled the outcome from three scenarios: (1) observed medium-term (5 year) annual mortality rates continue to the long term (20 years); (2) long-term annual mortality in retreatment responders falls to that of the general population while retreatment non-responders continue at the medium-term mortality; (3) long-term annual mortality in retreatment non-responders is the same as control group non-responders (i.e., the increased treatment-related medium mortality “wears off”).

**Results:**

The mean differences in life expectancy over 20 years with interferon versus control in the first, second, and third scenarios were -0.34 years (95% confidence interval (CI) -0.71 to 0.03), -0.23 years (95% CI -0.69 to 0.24), and -0.01 (95% CI -0.3 to 0.27), respectively. The life expectancy was always lower in the interferon group than in the control group in scenario 1. In scenario 3, the interferon group had a longer life expectancy than the control group only when more than 7% in the interferon group achieved SVR.

**Conclusions:**

SVR may be a good prognostic marker but does not seem to be a valid surrogate marker for assessing HCV treatment efficacy of interferon retreatment. The SVR threshold at which retreatment increases life expectancy may be different for different drugs depending upon the adverse event profile and treatment efficacy. This has to be determined for each drug by RCTs and appropriate modelling before SVR can be accepted as a surrogate marker.

## Introduction

### Disease prevalence, mode of transmission, and natural history of acute and chronic hepatitis C viral infection

Hepatitis C viral (HCV) infection affects 2% to 3% of the world’s population. This means that about 160 million people worldwide have chronic HCV infection[1]. HCV is transmitted by parenteral routes. Risk factors for transmission include parenteral drug abuse[2], transfusion of infected blood[3], sexual intercourse with infected individuals[4], perinatal transmission from mother to child[5], unhygienic tattooing practices[6], and occupational exposure to the blood of HCV infected patients[7]. Once infected, approximately 50% to 95% of patients have persistent HCV RNA in their blood, i.e., develop a chronic HCV infection [8–10] depending upon the genotype of the HCV[9].

The main complication associated with chronic HCV infection is damage to the liver leading to cirrhosis, decompensated liver disease, or hepatocellular carcinoma. Chronic hepatitis C is a slowly progressive disease. Liver-related morbidity and mortality, if they occur, usually happen 15 to 20 years after initial infection[11]. Approximately 1% to 39% of patients who develop chronic HCV infection develop cirrhosis after a period of 7 to 30 years[11–14]. Every year a proportion of patients with HCV-related cirrhosis who are referred to hospital with cirrhosis die (4%), develop liver failure manifested as ascites (3%), jaundice (2%), gastrointestinal bleeding (1%), or develop hepatocellular carcinoma (HCC) (4%)[15].

## Treatment

Various drugs have been used with the aim of eradicating chronic HCV infection, thereby preventing all the complications related to chronic HCV infection and subsequent cirrhosis. These include interferon (including pegylated interferon), directly acting anti-viral treatments (ribavirin; protease inhibitors such as telaprevir, bocerpevir), or a combination of the above drugs[16]. In patients with significant fibrosis experts currently recommend a combination of telaprevir or boceprevir, peginterferon, and ribavirin if the patient has genotype 1 or pegylated interferon and ribavirin for all other genotypes [16].

### How is efficacy of antivirals against hepatitis C assessed?

The recommendations on treatment for chronic HCV infection are based on the absence of detectable HCV RNA in the blood at the end of treatment and at 24 weeks after the end of treatment (sustained virological response (SVR)). Some consider SVR as virological ‘cure’ based on the belief that eradicating the virus from the blood prevents development of cirrhosis and subsequent complications. Observational studies have shown that patients who attain SVR have better survival and lower incidence of HCC than those who do not develop SVR[17–20]. The US Food and Drug Administration (FDA) approves drugs used in the treatment of chronic HCV on the basis of SVR[21]. Thus, SVR is widely believed to be the most important outcome in assessing the efficacy of treatments for patients with chronic HCV. 

However, achieving SVR may come at the price of exposing the patients to the adverse events of the drug(s) and it is necessary to balance the benefits (cure from chronic HCV) and harms (adverse events and adverse effects; time; costs to individual and society) of the antiviral intervention. The best way to assess the benefits and harms of interventions is through properly conducted systematic reviews of randomised clinical trials with low risk of systematic errors (bias) and of random errors (the effect of chance)[22] that assess clinical and patient-relevant outcomes such as mortality and morbidity directly.

### Evidence from a systematic review of re-treatment of non-responders and relapsers with interferon

A recent Cochrane systematic review of interferon retreatment for patients who did not respond to initial interferon therapy or relapsed after a period of virological response showed that patients receiving a second course of interferon alone are 18 times more likely to achieve SVR than those who received no treatment (3.3% versus 0.2%) [23]. However, paradoxically there was a 41% (95% confidence limits 2% to 95%) increase in mortality in patients receiving the second course of interferon as compared with no-intervention controls after an average follow-up period of 5 to 6 years (9.5% versus 6.7% mortality) based on information from trials with low risk of bias[23]. 

### Purpose of this study

The contrast between the results of randomised clinical trials which show no benefit of retreatment with interferon for HCV despite improvement in SVR and observational studies which show that patients who obtain SVR fare better is striking. As randomised clinical trials are considered to be a superior source of evidence, it appears that interferon is effective in achieving SVR, yet interferon is associated with a higher mortality in the medium term (5 to 6 years) when used for retreatment. The implication is that SVR is not a predictor of mortality in this group of patients. However, one could argue that the survival benefit of achieving SVR may only be evident after further 10 years to 15 years follow-up and that no such long-term data is available.

While the best way to determine the long-term outcome of treatment for patients with chronic HCV infection is a randomised clinical trial with long-term follow-up, there is no trial that has followed patients for such periods of time. In the absence of such information, decision modelling provides a means to incorporate the existing data with other information (including expert opinion), to determine the likely longer-term outcomes[24]. Sensitivity analysis can then be used to test the robustness of the conclusion to model assumptions. 

For this study, we employed a simple decision tree model to combine the medium-term mortality evidence from the systematic review with various assumptions as to the longer-term mortality. By making various assumptions as to the long-term mortality, we determined whether it was possible for SVR to result in better clinical outcomes over the long term (20 years), despite the poorer medium-term (5 years) outcomes in patients retreated with interferon monotherapy. Given the known medium-term data, if interferon monotherapy results in superior outcomes only under implausible assumptions for the longer-term mortality, this analysis would suggest that SVR is an inappropriate surrogate marker for survival in assessing the efficacy of a second course of interferon in patients who are non-responders or relapsers following the initial interferon intervention.

The findings of this research may be applicable to all non-responders and relapsers to antiviral therapy, who form more than 20% of patients with chronic HCV infection with the best treatments available[16]. In other words, approximately 30 million people may be affected by the results of this analysis. 

## Methods

### Decision tree model

**Figure 1:**
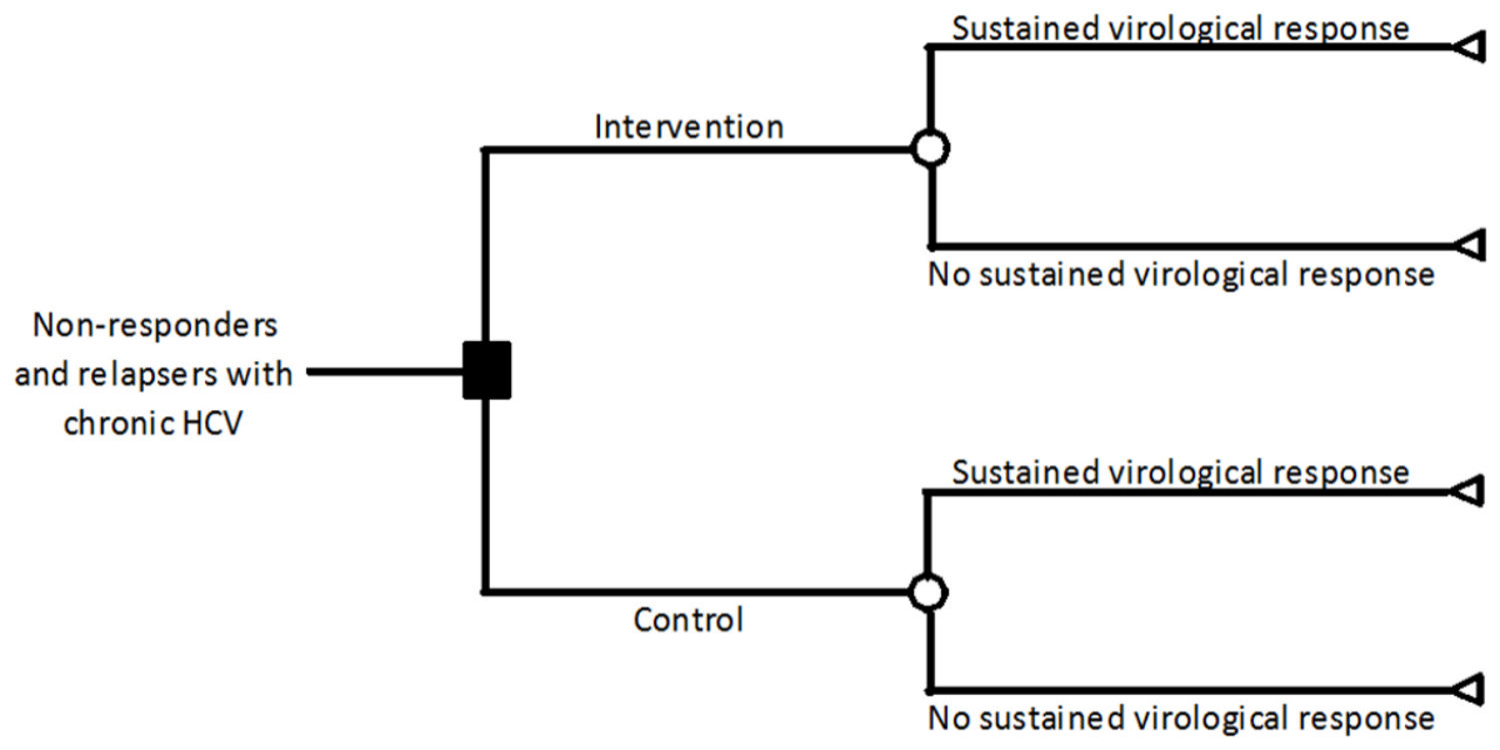
Decision tree. This figure shows the possible pathway followed by a patient with chronic hepatitis C infection, who does not respond to interferon monotherapy.

We created a simple decision tree model to illustrate the possible outcomes for a patient with chronic HCV infection (Figure 1). The software used was Microsoft Excel 2010. Patients with chronic HCV with failed first course of antiviral intervention undergo either intervention or no treatment (control) (the decision denoted by the blue square). Under intervention or control, there is a probability of the patient achieving SVR (denoted by the chance nodes - green circles). At the terminal nodes (denoted by the red triangles), the outcome is survival calculated as a simple two state Markov chain (alive or dead) with transition probabilities equal to the annual probability of death as described below. The base patient assumes a 50 year old male relapser or non-responder over a 20 year time horizon. For purposes of this analysis, medium-term mortality is the mortality in the first five years and long-term mortality is from years 5 to 20.

### Probabilities

The probabilities of the SVR and the five-year mortality were obtained from the Cochrane systematic review on ‘Interferon for interferon non-responding and relapsing patients with chronic hepatitis C’.[23] In the no-intervention control group, the SVR was 0.02% and the five-year mortality was 6.72%. The corresponding SVR and the five-year mortality in the interferon group were 3.31% and 9.47% based on risk ratios of 14.73 and 1.41 for SVR and five-year mortality, respectively[23]. Five-year mortality figures were converted to annual probabilities of death. Probability of SVR with interferon and mortality with interferon is expressed as the baseline (i.e., control) risk multiplied by the risk ratio. Background mortality for the general population was based on life expectancy tables for the UK by age [25] (Table 1).

**Table 1:** Model variables and parameters.

**Variable**	**Base case value**	**Distribution**	**Parameters**	**Source/Notes**
Probability of sustained virological response (SVR) in control group	0.02%	Beta	Alpha = 1 Beta = 559	Koretz et al.[23]
Log risk ratio (SVR: interferon versus no-intervention control)	2.9209	Normal	Mean = 2.9209 Standard deviation = 1.0255	Koretz et al.[23] Note this yields a base case probability of SVR in the interferon group of 3.31%
5-year probability of death in no-intervention control group	6.72%	Beta	Alpha = 57 Beta = 791	Koretz et al.[23] Annual probability therefore = 1.40% (= SR_c_ in Table 2)
Log risk ratio (death: interferon versus no-intervention control)	0.3424 (0.1649)	Normal	Mean = 0.3424 Standard deviation = 0.1649	Koretz et al.[23] Note this yields a base case 5 year probability of death with interferon = 9.47%, annual probability = 2.01% = SR_i_ in Table 2
Annual all-cause mortality by age	0.34% for 50 year male; 0.23% for 50 year female; 0.09% for 30 year male; 0.04% for 30 year female; 2.17% for 70 year old male; 1.41% for 70 year old female	Constant	Not applicable	Interim life tables (based on all reported deaths in the UK)[25], the sample size is assumed large enough for the standard error to be zero
Discount rate	3.50%	Constant	Not applicable	HM Treasury Green Book[26]

### Analysis

The outcome is the expected life years accrued per patient over a period of 20 years. We divided mortality into two periods based on the known medium-term mortality and the unknown long-term mortality. For years 0-4, the mortality from the Cochrane review was used. Beyond this we explored the following scenarios, which we considered the worst-case (scenario 1), the moderate-case (scenario 2), and the best-case (scenario 3) for use of SVR as a surrogate marker of mortality.

1. Mortality in both intervention and control groups continues at the same annual rate for years 5 to 19.
2. Mortality in patients with SVR (achieved on retreatment) falls to that of the general population for years 5 to 19, whilst patients with no SVR continue with an annual mortality risk similar to the rate over the first 5 years. This is an assumption producing a moderate survival benefit to SVR patients because some of these patients may have other risk factors such as intravenous drug abuse and associated behavioural problems such as chronic alcoholism which may increase their mortality.
3. Mortality in patients with SVR (achieved on retreatment) falls to that of the general population for years 5 to 19, whilst patients with no SVR continue with an annual mortality risk at the rate for no SVR in the control group. This is an assumption producing an extreme survival benefit to SVR patients because the assumption here is that the harmful effects of the interferon in patients without SVR have completely worn off while the annual mortality risk at the rate for SVR is that of the general population.

The death rates in the scenarios are summarised in Table 2. For each scenario, we have reported the expected life years accrued by interferon and control patients, the increment, associated 95% confidence interval, and the probability that interferon group had longer life expectancy than the control group. We also calculated the probability that the interferon group was better than the control group at different hypothetical SVR proportions in the interferon group. We performed a threshold analysis for the SVR proportions in the interferon group to explore whether there is a critical value at which life years gained in the interferon group was longer than in the control group. Life years accrued after the first year were discounted at 3.5% per annum, the currently recommended rate by UK Government[26]. 

**Table 2:** Summary of scenarios.

Scenario:		1		2		3	
		Annual probability of mortality		Annual probability of mortality		Annual probability of mortality	
		Years 0-4	5-19	Years 0-4	5-19	Years 0-4	5-19
Interferon	SVR	SR_i_	SR_i_	SR_i_	LR_p_	SR_i_	LR_p_
	No SVR	SR_i_	SR_i_	SR_i_	SR_i_	SR_i_	SR_c_
Control	SVR	SR_c_	SR_c_	SR_c_	LR_p_	SR_c_	LR_p_
	No SVR	SR_c_	SR_c_	SR_c_	SR_c_	SR_c_	SR_c_

where SVR = sustained virological response, SR_i,_ and SR_c_ = annual risk of death in interferon and control group as per systematic review; LR_p_ = annual risk of death in general population (as per notes in Table 1)

For the third scenario (long-term mortality in the SVR group falls to the same as the general population and the long-term mortality in the no SVR group falls to the same as the short-term mortality in the control group, i.e., the increased short-term mortality because of interferon treatment wears off), we also calculated the probability that the interferon group was better than control group at different odds ratios of long-term mortality in the no SVR group compared to the general population. This was done to account for the possibility of increased liver-related mortality in patients who did not achieve SVR which would take up to 20 years to manifest. For the same scenario, we performed a threshold analysis for the mortality in the no SVR group to explore whether there was a critical value of odds ratio at which life years gained in the interferon group was longer than in the control group. We also performed a two-way sensitivity analysis to identify the relationship between the probability of achieving SVR in the interferon group and the probability of long-term annual mortality rates in the no SVR group. We also repeated the analysis in the second scenario in patients of different sex and ages (50 year old female; 30 year old male; 30 year old female; 70 year old male; 70 year old female). 

To take account of uncertainty in mortality and SVR rates, probability distributions were assigned to all inputs (Table 1) and a Monte Carlo simulation of 5,000 iterations was conducted for each analysis.

## Results

The probabilities used in the decision tree are shown in Table 1. 

### Life expectancy

**Figure 2:**
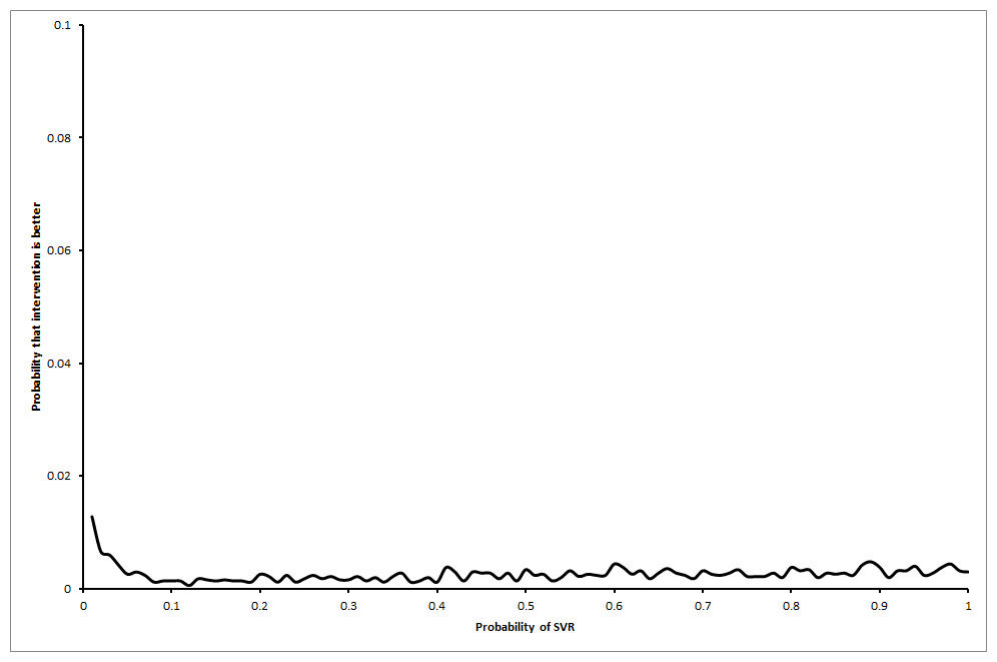
Probability that interferon retreatment is better at different sustained virological response (SVR) proportions in the interferon group (scenario 1). The chart shows that the probability of interferon retreatment resulting in better life expectancy than no-intervention control group is always below 2% if the long-term mortality in the two groups continued at the same rates as the short-term mortality (scenario 1).

**Figure 3:**
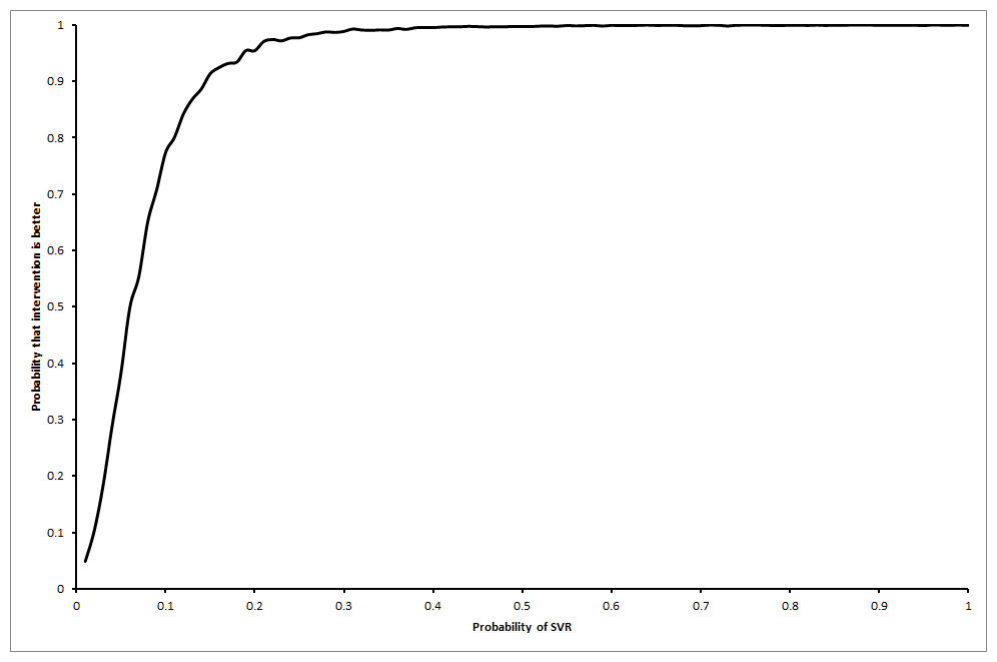
Probability that interferon retreatment is better at different sustained virological response (SVR) proportions in the interferon group (scenario 3). The chart shows that the probability of the interferon retreatment group having a longer life expectancy than the no-intervention control group increases as the proportion of patients with SVR in the interferon retreatment group increases. This is only true if the patients with no SVR in the interferon retreatment group had long-term mortality at the same rates as the medium-term mortality in the control group (i.e., the increased mortality in the medium-term due to interferon retreatment wears off) while those in the SVR group had a long-term mortality at the same rate as the general population (scenario 3).

**Figure 4:**
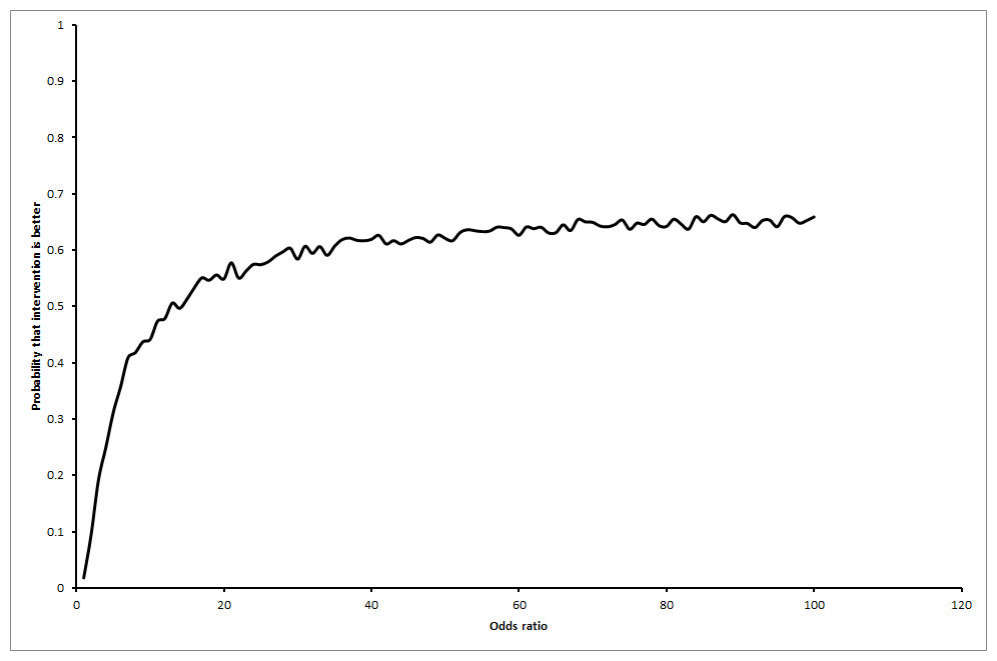
Probability that interferon retreatment is better at different odds ratio of long-term mortality in the no SVR group compared to the general population (scenario 3). This chart shows that the probability that interferon retreatment results in longer life expectancy compared to the no-intervention control group increases as the odds ratio of long-term mortality in the no-SVR group increases compared to the general population.

The life expectancy was shorter in the interferon group than the control group in all the scenarios for the base patient represented by a 50 year old male as shown in Table 3. The mean difference in life expectancy at 20 years between the interferon group and the control group in the first scenario was -0.34 years (95% CI -0.71 to 0.03), in the second scenario was -0.23 years (95% CI -0.69 to 0.24), and in the third scenario was -0.21 (95% CI -0.74 to 0.32). The probability that the interferon group has longer life expectancy than control group was 1.6% in the first, 11.4% in the second, and 27.9% in the third scenarios. The probability of interferon resulting in longer life expectancy than control was always below 2% in the first scenario irrespective of the proportion of patients who achieved SVR (Figure 2). As regards to the third scenario, the probability that interferon results in longer life expectancy than no intervention increases as the proportion of patients with SVR in the interferon group increases (Figure 3). For the third scenario, the probability that interferon results in longer life expectancy than no intervention increases as the odds ratio of long-term mortality in the no-SVR group compared with general population increases, but did not reach beyond 70% even at odds ratios of 100 (Figure 4). 

**Table 3:** Results - Life years gained in interferon retreatment versus no-intervention control, increment and 95% confidence intervals (CI).

**Scenario**	**Interferon**	**Control**	**Difference**	**Probability that interferon results in longer life expectancy**
Scenario 1: 30 years old male	11.75 (95% CI 11.18 to 12.32)	12.1 (95% CI 11.67 to 12.53)	-0.35 (95% CI -0.73 to 0.03)	1.30%
Scenario 2: 30 years old male	11.86 (95% CI 11.2 to 12.52)	12.09 (95% CI 11.66 to 12.52)	-0.23 (95% CI -0.72 to 0.26)	10.92%
Scenario 3: 30 years old male	12.08 (95% CI 11.56 to 12.61)	12.1 (95% CI 11.66 to 12.54)	-0.01 (95% CI -0.32 to 0.29)	27.58%
Scenario 1: 30 years old female	11.58 (95% CI 10.98 to 12.17)	11.93 (95% CI 11.47 to 12.38)	-0.35 (95% CI -0.73 to 0.03)	0.98%
Scenario 2: 30 years old female	11.72 (95% CI 11.01 to 12.43)	11.93 (95% CI 11.47 to 12.39)	-0.21 (95% CI -0.74 to 0.32)	13.08%
Scenario 3: 30 years old female	11.93 (95% CI 11.38 to 12.49)	11.93 (95% CI 11.48 to 12.38)	0 (95% CI -0.33 to 0.34)	32.08%
Scenario 1: 50 years old male	11.41 (95% CI 10.8 to 12.01)	11.74 (95% CI 11.26 to 12.22)	-0.34 (95% CI -0.71 to 0.03)	1.58%
Scenario 2: 50 years old male	11.52 (95% CI 10.86 to 12.18)	11.75 (95% CI 11.27 to 12.23)	-0.23 (95% CI -0.69 to 0.24)	11.36%
Scenario 3: 50 years old male	11.74 (95% CI 11.2 to 12.28)	11.75 (95% CI 11.28 to 12.23)	-0.01 (95% CI -0.3 to 0.27)	27.86%
Scenario 1: 50 years old female	11.52 (95% CI 10.92 to 12.11)	11.87 (95% CI 11.4 to 12.33)	-0.35 (95% CI -0.73 to 0.03)	1.54%
Scenario 2: 50 years old female	11.64 (95% CI 10.98 to 12.3)	11.87 (95% CI 11.41 to 12.32)	-0.23 (95% CI -0.7 to 0.25)	11.48%
Scenario 3: 50 years old female	11.85 (95% CI 11.3 to 12.4)	11.86 (95% CI 11.4 to 12.33)	-0.01 (95% CI -0.31 to 0.3)	29.50%
Scenario 1: 70 years old male	10.78 (95% CI 10.13 to 11.43)	11.11 (95% CI 10.56 to 11.66)	-0.33 (95% CI -0.69 to 0.04)	1.02%
Scenario 2: 70 years old male	10.8 (95% CI 10.17 to 11.43)	11.11 (95% CI 10.56 to 11.65)	-0.31 (95% CI -0.65 to 0.03)	0.98%
Scenario 3: 70 years old male	10.99 (95% CI 10.42 to 11.56)	11.11 (95% CI 10.55 to 11.67)	-0.12 (95% CI -0.3 to 0.06)	0.98%
Scenario 1: 70 years old female	10.81 (95% CI 10.18 to 11.45)	11.14 (95% CI 10.6 to 11.69)	-0.33 (95% CI -0.7 to 0.04)	1.30%
Scenario 2: 70 years old female	10.88 (95% CI 10.27 to 11.49)	11.15 (95% CI 10.61 to 11.69)	-0.27 (95% CI -0.6 to 0.06)	3.08%
Scenario 3: 70 years old female	11.06 (95% CI 10.53 to 11.6)	11.14 (95% CI 10.61 to 11.68)	-0.08 (95% CI -0.2 to 0.04)	5.38%

Scenario 1, 2, and 3 are shown in table 2.

### Threshold analyses

**Figure 5:**
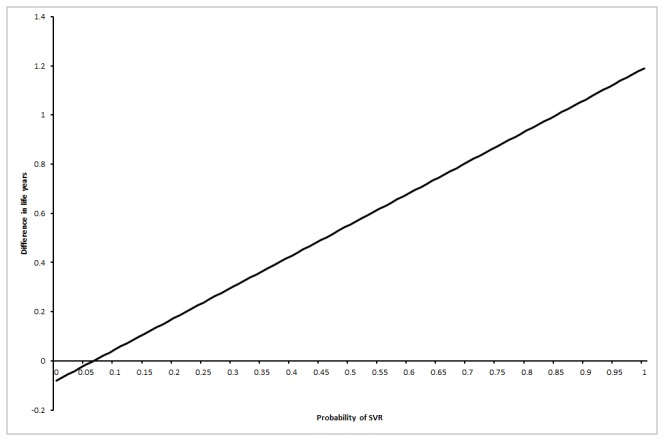
Difference in life expectancy between the intervention groups at various proportions of sustained virological response (SVR) in the interferon retreatment group (scenario 3). This chart shows that the interferon retreatment group had a longer life expectancy than the no-intervention control group if the proportion of patients who achieved SVR was 0.07 or above in the interferon retreatment group. This assumes that the patients with no SVR have a long-term mortality rate comparable to the medium-term mortality rate in the control group (i.e., the increased mortality in the medium-term due to treatment wears off) and those in the SVR group have a long-term mortality rate comparable to the general population (scenario 3).

**Figure 6:**
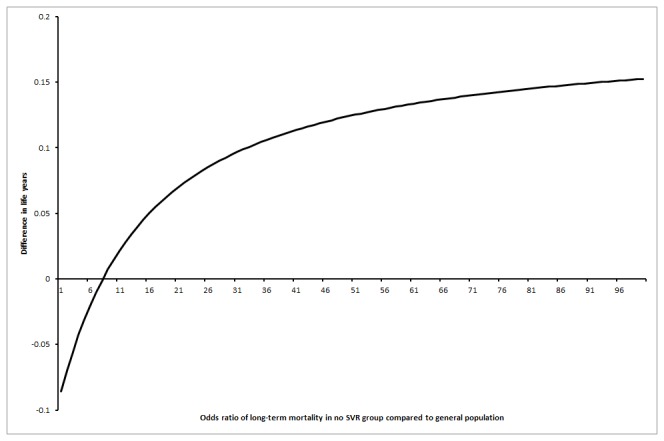
Difference in life expectancy between the intervention groups at different odds ratios of long-term mortality rates in no sustained virological response (SVR) group (scenario 3). This chart shows that the interferon retreatment group has a longer life expectancy than the no-intervention control group if the odds ratio of long-term mortality was 7.00 in the no SVR group compared to the general population. This also assumes that the patients in the SVR group had a long-term mortality rate similar to the general population (scenario 3).

In the first scenario, life expectancy was always lower in the interferon retreatment group irrespective of the proportion of patients who achieved SVR, i.e., no threshold level was reached. As regards to the third scenario, the interferon retreatment group had longer life expectancy than the control group when the proportion of SVR in the interferon group was more than 7% (Figure 5). This is an unlikely scenario according to the Cochrane review [23]. For the third scenario, the interferon retreatment group also had longer life expectancy than the control group if the odds ratio of long-term mortality in the no-SVR group was at least 9.00 compared to the general population (Figure 6). This is an unlikely scenario.

### Two-way sensitivity analysis

**Figure 7:**
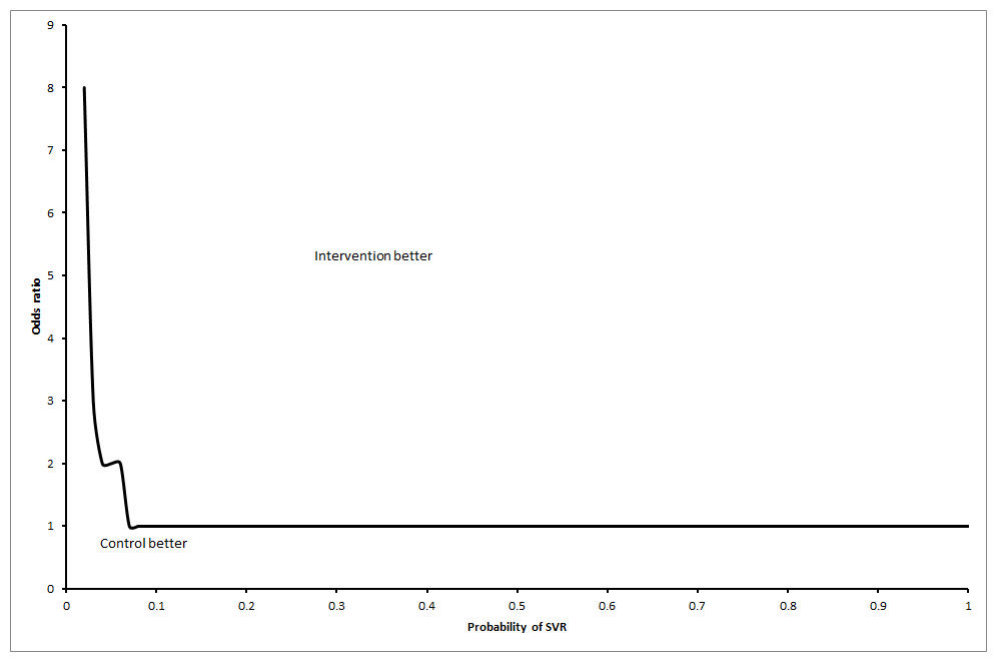
Two-way sensitivity analysis. The two-way sensitivity analysis of the odds ratios of long-term mortality in the no-SVR group compared to the general population versus the probability of SVR in the interferon group showed that in a 50 year old male, the interferon retreatment group had longer life expectancy than the no-intervention control group provided that the long-term mortality in the no-SVR group was higher than the general population. This assumes that the patients with no SVR have long-term mortality rate at the same rate as medium-term mortality in control group (i.e., the increased mortality in the medium-term due to treatment wears off), and if those in the SVR group had long-term mortality rates similar to the general population (scenario 3).

The two-way sensitivity analysis of the odds ratios of long-term mortality in the no-SVR group of the interferon retreatment group compared to the general population versus the probability of SVR in the interferon retreatment group showed that in a 50 year old male, the interferon group has longer life expectancy than the no intervention control group provided that the long-term mortality in the no SVR group is higher than that in the general population (Figure 7). 

### Change in sex and age

The results did not change for different ages and sex (Table 3). The threshold analysis revealed that the threshold of achieving SVR for the interferon group which resulted in longer life expectancy than in the control group was lower for a younger patient compared to an older patient and for a female compared to a male patient (Table 4), i.e., a lower level of achieving SVR would be acceptable for the interferon group in a younger patient compared to an older patient and for a female compared to a male patient. 

**Table 4:** Threshold analysis for sustained virological response (SVR) proportions in the interferon retreatment group and long-term mortality rates in the no SVR group.

**Age in years**	**Sex**	**Proportion of SVR in the interferon retreatment group**	**Odds ratio of mortality in the no-SVR group compared to the general population**
30	Male	6%	36.00
30	Female	6%	61.00
50	Male	7%	9.00
50	Female	6%	12.00
70	Male	Not reached†	4.00
70	Female	Not reached†	4.00

^†^ Probability of SVR was tested between 0 and 1.

### Discount rate

There was no noticeable change in the results when the discount rate was altered. 

## Discussion

### Main findings

This study has shown that SVR does not seem to be an appropriate surrogate outcome in non-responders and relapsers with chronic HCV infection retreated with interferon monotherapy. Despite achieving higher rates of viral clearance from the blood at 24 weeks after finishing retreatment, the second course of treatment resulted in shorter life expectancy compared with no treatment. The probability that the interferon retreatment resulted in improved long-term life expectancy, i.e., resulted in survival benefit, compared with the no-intervention group was less than 32.1% (less than 1 in 3 chance) in any of the different scenarios that we investigated. Chronic hepatitis C is a slowly progressive disease and liver-related morbidity and mortality are likely to occur after 15 to 20 years of having the disease[11]. However, we assumed that patients with no SVR had increased mortality in the medium term. This assumption would tend to favour SVR as a surrogate outcome. Even with this extreme assumption, the interferon patients who had higher SVR proportions had shorter life expectancy. Thus, one cannot consider SVR as a good or valid surrogate outcome for mortality for this group of patients retreated with interferon monotherapy. Although the two-way sensitivity analysis revealed that the interferon group has longer life expectancy than the no intervention control group, the extreme assumptions favouring SVR makes this finding unreliable.

### Applicability

This study is applicable only in patients with chronic HCV infection who did not respond to or relapsed following initial intervention containing interferon. The adverse events related to a specific treatment may differ to other treatments and hence the threshold levels at which interferon monotherapy retreatment becomes beneficial to patients may not be applicable to other groups of drugs. If SVR is to be used as an outcome in non-responders or relapsers with different first courses of treatment, then SVR must be validated as a surrogate outcome for that drug without assuming automatically that SVR is a valid surrogate outcome. 

As mentioned earlier, in patients with significant fibrosis experts currently recommend a combination of telaprevir or boceprevir, peginterferon, and ribavirin if the patient has genotype 1 or pegylated interferon and ribavirin for all other genotypes[16]. The proportion of patients who develop SVR is significantly higher with these drugs[27,28]. Whether the findings of this research can be extended to treatment naïve patients, particularly in those receiving telaprevir or boceprevir, or indeed newer drugs in the near future is not clear, as the SVR proportions are substantially higher with first treatment than the SVR observed with interferon retreatment. However, one has to carefully consider whether SVR is a suitable surrogate for treatment efficacy in treatment naïve patients and whether treatments such as telaprevir or boceprevir based on SVR might actually cause more harm by mechanisms other than SVR itself, for example, by altering host immunity or by effects on the replication of non-viral cells. Thus it is important to validate the use of SVR as a surrogate marker in different clinical settings and with different therapeutic regimens. The present study is not applicable to patients with acute HCV infection or acute or chronic infections of other chronic viral liver diseases.

### Strengths and weaknesses

#### All-cause mortality compared to liver-related mortality

We chose all-cause mortality rather than liver-related mortality as our patient-relevant outcome. Although SVR is assumed only to affect liver-related mortality, the risk of death with and without treatment due to any cause is more important for the patient. In addition all-cause mortality allows the success of treatment of chronic HCV to be compared with that of treatment of other diseases in which liver-related mortality is not a relevant outcome. This, in turn, allows appropriate use of limited financial and clinical resources. Liver-related mortality as an outcome in isolation has little significance unless all the effects of treatments are completely understood and there is robust evidence that the treatment does not have any other adverse effects that may influence mortality. Furthermore, liver-related mortality is likely to be a more biased outcome compared to all-cause mortality [22][23].

#### Sources of information

While several observational studies have shown that there is an improvement in survival by achieving SVR[17,19,20,29], none of the randomised clinical trials or systematic reviews of such trials have shown that this is the case[30,31]. This discrepancy could be due to a short follow-up period in the randomised clinical trials or because of selection bias in the observational studies. There was clear selection bias in some observational studies with patients with co-morbidities not receiving interferon treatment while those without co-morbidities or concerns related to adverse events of treatment were given interferon treatment[17,19]. Historical cohorts were also used[18,20]. Such information is known to be unreliable and the results are influenced by selection bias[22,32]. There is no evidence that the information from such observational studies which include patients with co-morbidities is better than indirect evidence from randomised clinical trials in patients without co-morbidities. Ideally, randomised clinical trials should be performed in patients with co-morbidities as well so that direct evidence can be obtained. 

Our present study is based on information from a systematic review of randomised clinical trials which provides the best available evidence[33]. As Cochrane systematic reviews are generally of a higher standard than other systematic reviews [34], we obtained the information from a Cochrane review[23]. Where information was not available from systematic reviews of randomised clinical trials, we made assumptions each time favouring SVR as a surrogate outcome and performed sensitivity analyses for a range of values for missing information. Unless new randomised clinical trials provide additional information this is currently the best-possible regarding interferon monotherapy used for retreatment.

#### Weaknesses of decision tree modelling

Decision modelling by definition involves simplification of situations and making some assumptions. The assumption that the patients achieving SVR have the same mortality as the general population is unlikely to be true. This is because these patients may have other risk factors such as intravenous drug abuse and associated behavioural problems such as chronic alcoholism which may increase their mortality compared to the general population. However, as mentioned previously, despite our choices of assumptions favouring SVR, the analysis shows that SVR is not a good and validated surrogate outcome for mortality. If SVR cannot be validated as a good surrogate outcome despite this ‘best-case scenario’, one can safely assume that SVR cannot be a good surrogate outcome in the real-life scenario or worst-case scenario. So, our conclusions will not change with plausible changes to these assumptions.

### Healthcare decision based on surrogate outcomes

There are costs related to any treatment. There may also be complications related to the treatment some of which might involve additional costs to resolve. It is important to maximise the benefits obtained from finite resources and this involves decision-making. The use of surrogate outcomes may decrease the costs and time required for an effective treatment to reach patients. However, the use of surrogate outcomes is acceptable only if they have been properly validated[30]. Our evaluation has shown that, SVR is an unvalidated surrogate outcome in non-responders or relapsers undergoing interferon retreatment[30] and thus is not helpful. It is possible, given the absence of evidence to the contrary, that this may be applicable with respect to other treatments in this group of patients considering the criteria for surrogate outcomes[35]. The threshold at which the long-term results of drug treatment outweigh any short-to medium-term adverse events may be different for different drugs. Accordingly validation of SVR is needed for each drug and drug combination, in every clinical setting, e.g., genotype of HCV, naïve, relapser and null responder patients, HIV co infected patients, cirrhotic patients and so on. Drugs which increase SVR may actually cause more deaths in some settings as evident in the Cochrane review[23]. This is not the first time that improvement in a surrogate outcome has actually resulted in more deaths or adverse outcomes[30]. Aprotinin decreases massive perioperative bleeding in patients undergoing high-risk cardiac surgery, but causes increased mortality when compared with other anti-fibrinolytics (BART trial)[36]. Aprotinin has now been withdrawn from the market for this reason[37]. If the BART trial had not assessed mortality as one of the outcomes it is highly likely that aprotinin would have been recommended for patients undergoing high-risk cardiac surgery on the basis of its ability to decrease massive perioperative bleeding. Risoglitazone decreases blood glucose and glycosylated haemoglobin but increases myocardial infarction and mortality in patients with diabetes[38]. If these outcomes were not measured, it is highly likely that risoglitazone would be recommended for diabetic patients based on its ability to decrease blood glucose levels and glycosylated haemoglobin. It is clear from the above examples that the use of *unvalidated* surrogate outcomes to justify use of therapy requires extreme caution. However, SVR has been accepted as a surrogate outcome by the medical and pharmaceutical community as well as the regulatory authorities without proper validation in every setting in which antiviral therapy is used. 

### Can SVR be used as a surrogate outcome for mortality and what are the implications?

There is no high-quality evidence to suggest that the long-term quality of life is better in patients who achieved SVR after retreatment. In the absence of such evidence of improvement in quality of life to compensate for the decreased life expectancy for retreatment, it is mandatory to inform such patients that a higher medium-term risk of death due to retreatment exists and that data for other treatments are not available. There is no scientific proof that interferon versus no intervention affects clinical outcomes positively[39]; that interferon plus ribavirin versus interferon affects clinical outcomes positively[31]; and that the newer antivirals combined with interferon plus ribavirin versus interferon plus ribavirin affects clinical outcomes positively[40]. HCV-related liver complications can occur even in patients who have achieved SVR and in addition, factors such as younger age, female sex, and low or no alcohol intake are common predictors of SVR and benign course of chronic HCV in untreated individuals[41–43]. Thus, SVR may not have a strong relationship with the decrease in liver-related complications at least in retreated patients with chronic HCV. 

A recent article criticised the use of glycosylated haemoglobin as a surrogate outcome for the assessment of efficacy of hypoglycaemic agents in diabetic patients[44]. Experts in evidence-based medicine do not recommend that surrogate outcomes be included in lists of outcomes except for exploration of mechanisms[32], although not withstanding this, SVR is the primary outcome in all therapeutic trial of antiviral therapy for HCV. Clinicians, researchers, drug companies and regulatory authorities should validate SVR as a surrogate outcome before using it as a primary outcome. An association of SVR with decreased mortality in observational studies is not enough to promote or prescribe a treatment because of the presence of confounding factors, which may themselves be causally related to the outcomes. Only a moderate proportion of patients with chronic HCV infection develop liver cirrhosis. The outcome in the entire group of treated patients is more important rather than the outcome in patients who have achieved SVR. While retreatment with interferon monotherapy may be applicable to only a small proportion of patients in the future, the principles of our present analyses are relevant for all interventions related to the treatment of patients with chronic HCV in the various clinical settings.

### Costs and cost-effectiveness of treatments

Throughout this discussion, we have not discussed the cost of the drug treatment. In the setting of state-funded healthcare systems, additional information regarding cost of the drug treatment and the quality of life of patients in the long-term are taken into account in determining whether treatment can be recommended. Such cost-effective analyses are likely to give wrong results (and hence wrong recommendations) if only unvalidated SVR is used in the modelling. Long term outcomes need to be evaluated.

## Conclusions

SVR is neither a good nor a validated surrogate marker for mortality in non-responders and relapsers with chronic HCV infection undergoing interferon reintervention after failed first course of interferon intervention. This observation may be different for different drugs and clinical settings. Validation of SVR should be performed for each individual drug and drug combination in each different clinical setting. Drug licensing agencies and national and international bodies should review the evidence that SVR is a suitable outcome for drugs used for re-treating such patients. Long-term outcomes of patients with chronic HCV infection involved in trials comparing treatments aimed at improving SVR need to be obtained and systematically re-evaluated to determine whether SVR is a valid surrogate outcome and the level of SVR at which the drug becomes beneficial to the patient. 

## References

[1] Lavanchy D (2011) Evolving epidemiology of hepatitis C virus. Clin Microbiol Infect 17: 107-115. doi:10.1111/j.1469-0691.2010.03432.x. PubMed: 21091831. 21091831

[2] Xia X, Luo J, Bai J, Yu R (2008) Epidemiology of hepatitis C virus infection among injection drug users in China: systematic review and meta-analysis. Public Health 122: 990-1003. doi:10.1016/j.puhe.2008.01.014. PubMed: 18486955. 18486955

[3] Kleinman SH, Lelie N, Busch MP (2009) Infectivity of human immunodeficiency virus-1, hepatitis C virus, and hepatitis B virus and risk of transmission by transfusion. Transfusion 49: 2454-2489. doi:10.1111/j.1537-2995.2009.02322.x. PubMed: 19682345. 19682345

[4] Tohme RA, Holmberg SD (2010) Is sexual contact a major mode of hepatitis C virus transmission? Hepatology 52: 1497-1505. doi:10.1002/hep.23808. PubMed: 20635398. 20635398

[5] Syriopoulou V, Nikolopoulou G, Daikos GL, Theodoridou M, Pavlopoulou I et al. (2005) Mother to child transmission of hepatitis C virus: rate of infection and risk factors. Scand J Infect Dis 37: 350-353. doi:10.1080/00365540510032105. PubMed: 16051571. 16051571

[6] Jafari S, Copes R, Baharlou S, Etminan M, Buxton J (2010) Tattooing and the risk of transmission of hepatitis C: a systematic review and meta-analysis. Int J Infect Dis 14: e928-e940. doi:10.1016/j.ijid.2010.03.019. PubMed: 20678951. 20678951

[7] Beltrami EM, Williams IT, Shapiro CN, Chamberland ME (2000) Risk and management of blood-borne infections in health care workers. Clin Microbiol Rev 13: 385-407. doi:10.1128/CMR.13.3.385-407.2000. PubMed: 10885983. 10885983 PMC88939

[8] Wawrzynowicz-Syczewska M, Kubicka J, Lewandowski Z, Boroń-Kaczmarska A, Radkowski M (2004) Natural history of acute symptomatic hepatitis type C. Infection 32: 138-143. doi:10.1007/s15010-004-3062-8. PubMed: 15188072. 15188072

[9] Lehmann M, Meyer MF, Monazahian M, Tillmann HL, Manns MP et al. (2004) High rate of spontaneous clearance of acute hepatitis C virus genotype 3 infection. J Med Virol 73: 387-391. doi:10.1002/jmv.20103. PubMed: 15170633. 15170633

[10] Beinhardt S, Aberle JH, Strasser M, Dulic-Lakovic E, Maieron A et al. (2012) Serum level of IP-10 increases predictive value of IL28B polymorphisms for spontaneous clearance of acute HCV Infection. Gastroenterology 142: 78-85. doi:10.1053/j.gastro.2011.09.039. PubMed: 22192885. 22192885

[11] Wiese M, Grüngreiff K, Güthoff W, Lafrenz M, Oesen U et al. (2005) Outcome in a hepatitis C (genotype 1b) single source outbreak in Germany--a 25-year multicenter study. J Hepatol 43: 590-598. doi:10.1016/j.jhep.2005.04.007. PubMed: 16237783. 16237783

[12] Seeff LB (2009) The history of the "natural history" of hepatitis C (1968-2009). Liver International 29 Suppl 1: 89-99. doi:10.1111/j.1478-3231.2008.01927.x. PubMed: 19207971. PMC4373556 19207971

[13] Kenny-Walsh E (1999) Clinical outcomes after hepatitis C infection from contaminated anti-D immune globulin. Irish Hepatology Research Group. N Engl J Med 340: 1228-1233. doi:10.1056/NEJM199904223401602. PubMed: 10210705. 10210705

[14] Rodger AJ, Roberts S, Lanigan A, Bowden S, Brown T et al. (2000) Assessment of long-term outcomes of community-acquired hepatitis C infection in a cohort with sera stored from 1971 to 1975. Hepatology 32: 582-587. doi:10.1053/jhep.2000.9714. PubMed: 10960453. 10960453

[15] Sangiovanni A, Prati GM, Fasani P, Ronchi G, Romeo R et al. (2006) The natural history of compensated cirrhosis due to hepatitis C virus: A 17-year cohort study of 214 patients. Hepatology 43: 1303-1310. doi:10.1002/hep.21176. PubMed: 16729298. 16729298

[16] Rosen HR (2011) Clinical practice. Chronic hepatitis C infection. N Engl J Med 364: 2429-2438. doi:10.1056/NEJMcp1006613. PubMed: 21696309. 21696309

[17] Maruoka D, Imazeki F, Arai M, Kanda T, Fujiwara K et al. (2012) Long-Term Cohort Study of Chronic Hepatitis C according to Interferon Efficacy. J Gastroenterol Hepatol 27: 291-299. doi:10.1111/j.1440-1746.2011.06871.x. PubMed: 21793911. 21793911

[18] Imai Y, Tamura S, Tanaka H, Hiramatsu N, Kiso S et al. (2010) Reduced risk of hepatocellular carcinoma after interferon therapy in aged patients with chronic hepatitis C is limited to sustained virological responders. J Viral Hepat 17: 185-191. doi:10.1111/j.1365-2893.2009.01163.x. PubMed: 19709362. 19709362

[19] Imazeki F, Yokosuka O, Fukai K, Saisho H (2003) Favorable prognosis of chronic hepatitis C after interferon therapy by long-term cohort study. Hepatology 38: 493-502. doi:10.1016/S0270-9139(03)80733-4. PubMed: 12883494. 12883494

[20] Tanaka H, Tsukuma H, Kasahara A, Hayashi N, Yoshihara H et al. (2000) Effect of interferon therapy on the incidence of hepatocellular carcinoma and mortality of patients with chronic hepatitis C: a retrospective cohort study of 738 patients. Int J Cancer 87: 741-749. doi:10.1002/1097-0215(20000901)87:5. PubMed: 10925370. 10925370

[21] US Food and Drug Administration (2011) FDA approves Incivek for hepatitis C. Available: http://www.fda.gov/NewsEvents/Newsroom/PressAnnouncements/ucm256299.htm. Accessed 24 October 2011

[22] Keus F, Wetterslev J, Gluud C, van Laarhoven CJ (2010) Evidence at a glance: error matrix approach for overviewing available evidence. BMC Med Res Methodol 10: 90. doi:10.1186/1471-2288-10-90. PubMed: 20920306. 20920306 PMC2959031

[23] Koretz RL, Pleguezuelo M, Arvaniti V, Baena PB, Ciria R et al. (2013) Interferon for interferon nonresponding and relapsing patients with chronic hepatitis C. Cochrane Database of Systematic Reviews: CD, 1: 003617 PubMed: 23440791. 10.1002/14651858.CD003617.pub2 PMC6599819 23440791

[24] Buxton MJ, Drummond MF, Van Hout BA, Prince RL, Sheldon TA et al. (1997) Modelling in economic evaluation: an unavoidable fact of life. Health Econ 6: 217-227. doi:10.1002/(SICI)1099-1050(199705)6:3. PubMed: 9226140. 9226140

[25] Office for National Statistics (2011) Interim Life Tables, 2008-2010. Available: http://www.ons.gov.uk/ons/publications/re-reference-tableshtml?edition=tcm%3A77-223324. Accessed 24 October 2011

[26] HM Treasury (2003) (updated 2011)) The Green Book. Appraisal and Evaluation in Central Government. Available: http://www.hm-treasury.gov.uk/d/green_book_complete.pdf. Accessed 24 October 2011

[27] Kong Y, Wang X, Shang Y, Schroder PM, Liang W et al. (2012) Efficacy and tolerability of telaprevir for chronic hepatitis virus C genotype 1 infection: a meta-analysis. PLOS ONE 7: e52158. doi:10.1371/journal.pone.0052158. PubMed: 23284915. 23284915 PMC3527389

[28] Chou R, Hartung D, Rahman B, Wasson N, Cottrell EB et al. (2013) Comparative effectiveness of antiviral treatment for hepatitis C virus infection in adults: a systematic review. Ann Intern Med 158: 114-123. doi:10.7326/0003-4819-158-2-201301150-00576. PubMed: 23437439. 23437439

[29] Ng V, Saab S (2011) Effects of a sustained virologic response on outcomes of patients with chronic hepatitis C. Clin Gastroenterol Hepatol 9: 923-930. doi:10.1016/j.cgh.2011.05.028. PubMed: 21699815. 21699815

[30] Gluud C, Brok J, Gong Y, Koretz RL (2007) Hepatology may have problems with putative surrogate outcome measures. J Hepatol 46: 734-742. doi:10.1016/j.jhep.2007.01.003. PubMed: 17316871. 17316871

[31] Brok J, Gluud LL, Gluud C (2010) Ribavirin plus interferon versus interferon for chronic hepatitis C. Cochrane Database of Systematic Reviews: CD: 005445 PubMed: 2009157716034976. 10.1002/14651858.CD005445 16034976

[32] Higgins J, Green S. Cochrane Handbook for Systematic Reviews of Interventions Version 5.1.0 [updated March 2011]. The Cochrane Collaboration, 2011. Available from www.cochrane-handbook.org.

[33] Atkins D, Best D, Briss PA, Eccles M, Falck-Ytter Y et al. (2004) Grading quality of evidence and strength of recommendations. BMJ 328: 1490. doi:10.1136/bmj.328.7454.1490. PubMed: 15205295. 15205295 PMC428525

[34] Jadad AR, Cook DJ, Jones A, Klassen TP, Tugwell P et al. (1998) Methodology and reports of systematic reviews and meta-analyses: a comparison of Cochrane reviews with articles published in paper-based journals. JAMA 280: 278-280. doi:10.1001/jama.280.3.278. PubMed: 9676681. 9676681

[35] Bucher HC, Guyatt GH, Cook DJ, Holbrook A, McAlister FA (1999) Users' guides to the medical literature: XIX. Applying clinical trial results A. How to use an Article measuring the effect of an intervention on surrogate end points Evidence-Based Medicine Working Group. JAMA 282: 771-778 10.1001/jama.282.8.771 10463714

[36] Fergusson DA, Hébert PC, Mazer CD, Fremes S, MacAdams C et al. (2008) A comparison of aprotinin and lysine analogues in high-risk cardiac surgery. N Engl J Med 358: 2319-2331. doi:10.1056/NEJMoa0802395. PubMed: 18480196. 18480196

[37] MHRA (2007) Aprotinin (Trasylol): suspension of UK marketing authorisations (licences). Available: http://www.mhra.gov.uk/Safetyinformation/Safetywarningsalertsandrecalls/Safetywarningsandmessagesformedicines/CON2033201. Accessed 3 September 2011

[38] Nissen SE, Wolski K (2007) Effect of rosiglitazone on the risk of myocardial infarction and death from cardiovascular causes. N Engl J Med 356: 2457-2471. doi:10.1056/NEJMoa072761. PubMed: 17517853. 17517853

[39] Myers RP, Regimbeau C, Thevenot T, Leroy V, Mathurin P et al. (2002) Interferon for interferon naive patients with chronic hepatitis C. Cochrane Database of Systematic Reviews: CD: 000370 PubMed: 12076394. 10.1002/14651858.CD000370 PMC7061493 12076394

[40] Lok AS, Gardiner DF, Lawitz E, Martorell C, Everson GT et al. (2012) Preliminary study of two antiviral agents for hepatitis C genotype 1. N Engl J Med 366: 216-224. doi:10.1056/NEJMoa1104430. PubMed: 22256805. 22256805

[41] Tohra SK, Taneja S, Ghosh S, Sharma BK, Duseja A et al. (2011) Prediction of sustained virological response to combination therapy with pegylated interferon alfa and ribavirin in patients with genotype 3 chronic hepatitis C. Dig Dis Sci 56: 2449-2455. doi:10.1007/s10620-011-1770-3. PubMed: 21706207. 21706207

[42] Poynard T, Bedossa P, Opolon P (1997) Natural history of liver fibrosis progression in patients with chronic hepatitis C. The OBSVIRC, METAVIR, CLINIVIR, and DOSVIRC groups. Lancet 349: 825-832. doi:10.1016/S0140-6736(96)07642-8. PubMed: 9121257. 9121257

[43] Pineda JA, Caruz A, Rivero A, Neukam K, Salas I et al. (2010) Prediction of response to pegylated interferon plus ribavirin by IL28B gene variation in patients coinfected with HIV and hepatitis C virus. Clin Infect Dis 51: 788-795. doi:10.1086/656235. PubMed: 20804372. 20804372

[44] Yudkin JS, Lipska KJ, Montori VM (2011) The idolatry of the surrogate. BMJ 343: d7995. doi:10.1136/bmj.d7995. PubMed: 22205706. 22205706

